# Histological and Proteomic Approaches to Assessing the Adrenal Stress Response in Common Dolphins (*Delphinus delphis*)

**DOI:** 10.3390/ani15192924

**Published:** 2025-10-09

**Authors:** Claudia Medina Santana, Orla Slattery, Jim O’Donovan, Sinéad Murphy

**Affiliations:** 1 Marine and Freshwater Research Centre, Department of Natural Resources and the Environment, School of Veterinary, Agriculture and Environmental Sciences, Atlantic Technological University, Dublin Road, H91 T8NW Galway, Ireland; orla.slattery@atu.ie; 2 Department of Analytical, Biopharmaceutical and Medical Science, School of Life Sciences, Atlantic Technological University, Dublin Road, H91 T8NW Galway, Ireland; 3 Department of Agriculture, Food and the Marine, Regional Veterinary Laboratory, Model Farm Road, T12 XD51 Cork, Ireland

**Keywords:** common dolphin, adrenal gland, chronic stress, acute stress, histology, FFPE tissue, conservation biology

## Abstract

**Simple Summary:**

Dolphins, like humans, rely on their adrenal glands to produce hormones that regulate stress. Yet, little is known about how these glands respond to long-term stress in wild populations. In this study, we examined adrenal glands from common dolphins (*Delphinus delphis*) stranded along the Irish coast. Animals that died suddenly from causes such as bycatch were compared with those that succumbed to infectious disease, representing chronic stress. Dolphins that experienced chronic stress had significantly larger adrenal cortices and higher cortex-to-medulla ratios, consistent with prolonged hormone production. We also tested a pilot method for extracting proteins from formalin-fixed, paraffin-embedded tissues and identified several stress-related proteins, some associated with the type of stress experienced. These findings demonstrate that combining tissue structure and protein analysis can reveal markers of chronic stress in dolphins and highlight the potential of archived material for improving conservation health assessments.

**Abstract:**

The adrenal glands are central to the stress response in cetaceans, yet their morphological and molecular changes under chronic stress remain poorly described. We investigated adrenal histology and protein composition in stranded common dolphins (*Delphinus delphis*) to assess whether post-mortem material can provide insights into stress physiology. Adrenal glands from 58 dolphins recovered along the Irish coast during a period of reported nutritional stress in the species were analyzed for adrenal mass, cortex-to-medulla (C:M) ratios, and cortical cell density. Additionally, two archival formalin-fixed, paraffin-embedded (FFPE) tissues were included in a pilot trial to assess the feasibility of protein extraction and mass spectrometry analysis. While adrenal mass did not differ significantly between stress types, chronically stressed dolphins exhibited significantly higher C:M ratios and cortical mass, consistent with cortical hypertrophy. Protein extraction from FFPE tissues was feasible, with the in-gel digestion method yielding more proteins (136) than the filter-aided sample preparation method (22). These findings demonstrate that histological and proteomic approaches can detect stress-related signatures in dolphins and highlight the potential of archival tissues for retrospective biomarker discovery.

## 1. Introduction

Stress in mammals has been defined as a physiological condition in which external or internal factors disrupt homeostasis, destabilizing the body’s regulatory systems and altering their normal functioning [1]. This destabilization rapidly triggers multiple physiological reactions, including heightened production and release of catecholamines, opioids, adrenocorticotropic hormone (ACTH), and glucocorticoids (GCs) [2]. 

A key component of this process is activation of the hypothalamus–pituitary–adrenal (HPA) axis, which regulates the secretion of hormones mediating the stress response [3]. The adrenal glands, small paired endocrine organs located atop the kidneys, play a central role. Each gland consists of an inner medulla and an outer cortex, the latter divided into the zona glomerulosa, zona fasciculata, and zona reticularis, each producing distinct steroid hormones [4]. ACTH, released from the pituitary gland, stimulates the adrenal cortex to secrete glucocorticoids such as cortisol and corticosterone [5]. These hormones regulate electrolyte balance, energy metabolism, and immune function, and their concentrations serve as indicators of the physiological stress response in cetaceans [6,7,8,9]. 

Prolonged ACTH stimulation can also produce macroscopic changes, including adrenal mass enlargement and cyst formation [6,10]. Adrenal pathology, including cortical hyperplasia, congestion, adrenalitis, and extramedullary hematopoiesis, has also been documented in rough-toothed dolphins (*Steno bredanensis*), supporting its link to stress-related processes [11]. Other indicators of stress include lipid depletion and hyperemia, which cause a darker adrenal cortex. These features have been observed in spinner and spotted dolphins (*Stenella* spp.) captured during fishing operations, consistent with acute stress responses [1]. Organochlorines and other endocrine-disrupting chemicals have been associated with impaired adrenal function—particularly due to polychlorinated biphenyl (PCB) exposure [12]—as well as with reduced immunity [13], and decreased reproductive success [14].

Histological studies have been widely applied to investigate stress-induced pathology in cetaceans. Chronic stress in odontocetes has been linked to hypertrophy (increased cell size) and hyperplasia (increased cell number) within the zona fasciculata of the adrenal cortex [15,16,17]. In stranded bottlenose dolphins (*Tursiops truncatus*), chronic disease was associated with increased adrenal mass, altered cortex-to-medulla ratios, and elevated numbers of epinephrine-producing cells relative to acutely stressed controls [18]. Following exposure to an oil spillbottlenose dolphins exhibited thinner adrenal cortices and a higher prevalence of adrenal and pulmonarydisease [19]. Endocrine assessments provide complementary insights. Cortisol levels in blubber have been used as indicators of stress in free-ranging cetaceans, with post-mortem concentrations reflecting both life history and mode of death [9,20]. Recently, a simplified blubber-extraction method developed for common dolphins (*Delphinus delphis*) has shown promise as a cost-effective tool for quantifying cortisol, though efficiency remains low [7]. A non-invasive approach has also been validated, using small, scraped skin samples to measure epidermal cortisol in bottlenose dolphins and beluga whales (*Delphinapterus leucas*), offering a low risk method for stress monitoring [8].

Although adrenal changes under chronic stress are less well-documented in marine mammals, terrestrial models offer valuable insight. In rodents, chronic stress leads to adrenal cortical and medullary hypertrophy, increasing glucocorticoid production [21]. Meanwhile, in mice, the combination of chronic stress with cardiovascular disease upregulated adrenal mitochondrial enzymes such as citrate synthase and oxoglutarate dehydrogenase (OGDH), reflecting increased metabolic activity [22]. Studies in golden hamsters showed that chronic infection can cause adrenal hypertrophy, lipid depletion, and necrosis, which are key features of adrenal exhaustion [23]. Additionally, research in both humans and rodents demonstrated that chronic systemic conditions like sepsis and obesity remodel the adrenal microenvironment through immune-endocrine interactions, ultimately altering hormone output [24].

Proteomics has emerged as a promising tool for assessing stress physiology. Blubber proteomic profiles have identified potential markers of chronic stress [25], highlighting the importance of integrating endocrine, transcriptomic, and proteomic approaches [26]. Establishing robust proteomic baselines is essential for the effective use of protein biomarkers in evaluating health status [27]. Candidate biomarkers include cytochrome P450 enzymes [28], heat shock proteins (e.g., HPS70) [29], and glutathione S-transferases [30]. These proteins offer valuable diagnostic insights into pollutant exposure, oxidative stress, and metabolic disruption, supporting both clinical assessments and conservation-focused applications [31,32].

Marine mammals are exposed to a range of anthropogenic and environmental stressors, including fishing activities, vessel traffic, oil and gas operations, other sources of underwater noise, and declining prey availability [33,34]. Recognizing the potential impacts of these stressors, the National Academies of Sciences, Engineering, and Medicine [35] highlighted the importance of evaluating their effects on physiological processes, vital rates, and overall population stability. The short-beaked common dolphin (*Delphinus delphis*) is the most abundant cetacean in the Northeast Atlantic and, globally, occurs across temperate to tropical waters [36,37]. At a species level it’s listed as Least Concern, but some subpopulations have a higher conservation concern, such as the Mediterranean subpopulation, which is classified as endangered [38]. In Europe, bycatch is the leading threat, disproportionately affecting common dolphins due to their abundance and foraging ecology [39,40]. Additional major stressors include exposure to pollutants and reduced prey availability [37,40]. Recent findings indicate a decline in the nutritional health of common dolphins in the Celtic Seas ecoregion, marked by increasing cases of emaciation and starvation-related mortality [41]. Additionally, high burdens of stomach parasites such as *Anisakis* spp. are frequently observed and often linked to poor nutritional state and gastric ulceration, underscoring the impact of ecological stressors on common dolphin health in the region [42]. Anthropogenic and environmental pressures may result in acute mortality or chronic sub-lethal effects, both of which manifest in physiological changes detectable post-mortem.

While histological and endocrine approaches have provided valuable insight into stress physiology in cetaceans, they capture only part of the picture. Proteomic analyses offer an opportunity to move beyond hormone levels and structural pathology, allowing identification of stress-related molecular signatures within the adrenal gland itself. Despite advances in blubber proteomics [25] and recent efforts to refine cortisol extraction from blubber [7] and skin [8], comparable protocols for adrenal tissue remain limited, particularly when working with paraffin-embedded samples from stranded animals. Developing a reliable method for protein extraction from these tissues would enable the detection of stress-related biomarkers directly within the primary endocrine organ mediating the stress response, bridging histological observations with molecular mechanisms and strengthening our ability to assess chronic stress in common dolphins.

Cetaceans display stress responses similar to those of other mammals and are typically classified as either acute or chronic. Acute stress arises from sudden events such as net entanglement, vessel collisions, intraspecific aggression, or acute infections, typically leading to rapid mortality before structural changes develop in the adrenal gland [3]. In contrast, chronic stress arises from prolonged disease (e.g., pneumonia, hepatitis) or repeated exposure to stressors, which allows time for detectable histological and functional alterations [18].

This study aims to (1) assess chronic stress pathology in the adrenal glands of common dolphins by adapting established histological methodologies [16,18] to detect hypertrophy, hyperplasia, and other lesions; (2) conduct a pilot study to develop a proteomic extraction protocol for formalin-fixed, paraffin-embedded (FFPE) adrenal tissues, following García-Vence et al. [43], and characterize the proteomic profile of the extracted samples; and (3) contribute baseline data integrating histological and proteomic approaches, providing new tools for assessing stress physiology in cetaceans and informing conservation strategies.

## 2. Materials and Methods

### 2.1. Sample Collection

Common dolphins were recovered along Irish coastlines (County Donegal to County Wexford) for postmortem examinations between 2017 and 2019. Necropsies were carried out by a qualified veterinary pathologist at the Regional Veterinary Laboratory in Cork, following the standardized necropsy protocol of the UK Cetacean Stranding Investigation Programme (CSIP). During necropsies, data on total body length (cm), sex, decomposition state, and nutritional status were recorded (see [44] for further information). Body length was measured by placing the carcass in ventral recumbency and using a measuring tape aligned parallel to the longitudinal body axis, from the tip of the upper jaw to the notch of the tail flukes [44]. Only animals not frozen prior to necropsy and in extremely fresh to moderate decomposition states were included in the current study. Causes of death (CODs) were determined based on collective findings from post-mortem investigations using diagnostic criteria established by the CSIP. CODs were classified into categories including infectious disease, bycatch, starvation, live stranding, and others (e.g., gas embolism, maternal separation, etc.). Sexual maturity in females was determined by the presence of one or more corpora scars (lutea or albicanta) on their ovaries [45]. For males, sexual maturity status was evaluated through histological analysis of gonadal tissue, following the methodology outlined in the work of Murphy et al. [46]. Nutritional status was assessed through external examination of the flanks around the dorsal fin, noting whether they appeared convex, flat, or concave, and by recording any indentation posterior to the head along the dorsal aspect [47]. Based on these observations, animals were classified as having good, moderate, or poor to very poor nutritional status [41]. For the present study, the left and right adrenal glands were collected and fixed in 10% neutral buffered formalin until further analysis.

The sample consisted of 31 males, 25 females, and two individuals of unknown sex, with body lengths ranging from 93–229 cm in males and 138.5–224 cm in females. Of the 58 dolphins, 39.7% (*n* = 23) were in a fresh decomposition state, 36.2% (*n* = 21) in slight decomposition, and 24.1% (*n* = 14) in moderate decomposition. Among individuals for whom sexual maturity status was assessed, 33 were classified as sexually mature and 23 as sexually immature. Regarding nutritional status, the sample included 43.1% (*n* = 25) in poor status, 43.1% (*n* = 25) in moderate status, and 13.8% (*n* = 8) in good nutritional status.

Based on cause of death, 19 individuals were classified as ‘chronically’ stressed, having died from infectious diseases indicative of prolonged stress, while 14 individuals were categorized as ‘acutely’ stressed, with deaths attributed to sudden stressors such as bycatch or physical trauma. The remaining individuals (*n* = 25) were classified as ‘other’ when the cause of death could not be clearly associated with either type—for example, in cases of live stranding, starvation or undetermined causes. A breakdown of the biological characteristics across stress categories is provided in Table 1.

### 2.2. Histological Examination

After a period of fixation of four to six years, the length, width, depth, and weight of each adrenal gland were recorded. Transverse slices, 2–3 mm thick, were taken perpendicular to the long axis at the midpoint of each gland, following previously detailed methods [16,18]. Sub-samples were then processed for histological analysis. Tissues were processed using a Leica TP1020 tissue processor (Leica Biosystems, Wetzlar, Germany) following a standard reagent sequence: fixation in 10% neutral buffered formalin, dehydration through a graded ethanol series (70, 80, 95, 100%) and clearing with Neo-clear (MilliporeSigma, St. Louis, MO, USA). After paraffin embedding, 5 μm thick sections were cut from each block and mounted on glass slides. The slides were then stained with hematoxylin and eosin (H&E) using an automated staining protocol on the Leica ST5010 Autostainer XL (Leica Biosystems, Wetzlar, Germany).

#### Imaging Technique and Measurements

**Figure 1:**
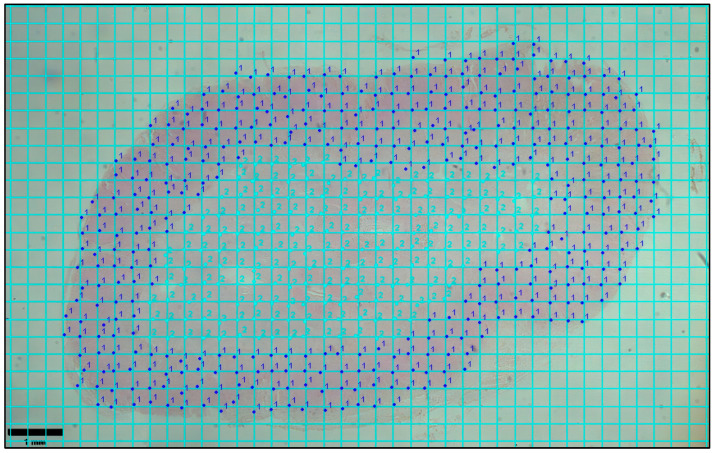
Cross-section of left adrenal gland from an acutely stressed common dolphin (*Delphinus delphis*) stained with hematoxylin and eosin. The section is overlaid with a grid consisting of 0.2 mm^2^ squares to facilitate point-counting analysis. Points labeled “1” indicate areas assigned to the adrenal cortex, while points labeled “2” indicate areas assigned to the adrenal medulla. Bar 1 mm.

Image-Pro^®^ v10.0.13 software (Media Cybernetics, Inc., Rockville, MD, USA) was used to capture and visualize each adrenal cross-section at a 12.5× magnification using an Olympus BX41 microscope (Olympus Corporation, Tokyo, Japan). The point-counting technique was subsequently applied to calculate the ratio between the cortical and medullary cross-sectional areas (after [3,16,18]). A grid with squares measuring 0.2 mm^2^ was applied to each cross-section image using the ImageJ software [48] (Figure 1). A smaller grid size, and thus a greater number of points, was chosen to enhance accuracy of the area percentage estimation [49]. Using the manual cell counter tool in Image J v1.53t software (National Institutes of Health, Bethesda, MD, USA), each point was classified as either cortex or medulla tissue to calculate the cortex-to-medulla ratio. Previous research indicated that cortico-medullary ratios remain consistent along the length of a dolphin’s adrenal gland, suggesting that measures taken from the mid-region reliably represent the entire gland [18].

Images of the secretory cells were captured at 400× magnification at three randomly chosen points along the cortex: one adjacent to the capsule, one near the medulla, and one at an intermediate point between the two, as described in [16]. Nuclei within secretory cells were manually counted in each image using the cell counter tool in Image J software. To enhance accuracy, the entire area of each image was used for cell counting. The number of counted nuclei cells was divided by the image area (in mm^2^) to calculate the cell density (nuclei/mm^2^) and the average of three images per gland was used to estimate the overall secretory cell density in the adrenal cortex. These data were used to assess cellular hypertrophy—indicated by a reduced cell density (nuclei concentration) alongside increased cell volume, reflecting cell enlargement—and cellular hyperplasia—characterized by an equal or higher concentration of cells (nuclei) combined with increased cell volume, indicating an increase in cell number [16]. The images also supported the evaluation of cellular abnormalities and overall cell condition through comparisons with reference samples of healthy adrenal tissue. Samples from dolphins in advanced decomposition and dolphins who were frozen prior to necropsy were not included in the cell count analysis, due to the compromised condition of the cells.

### 2.3. Statistical Analysis

Shapiro–Wilk tests were performed prior to all analyses to assess the normality of the datasets. Outliers were identified visually using boxplots (Tukey’s method, 1.5× IQR rule) for each variable. Values beyond the whiskers were considered outliers and removed prior to calculation of statistical analyses. Further, to assess differences between the left and right adrenal glands, paired *t*-tests were conducted on the normally distributed cortex-to-medulla ratio (C:M), adrenal gland mass (AM), and cortical cell density (CCD) datasets. Only the left adrenals of each dataset (normally distributed) were used in the following tests.

Bartlett’s test was employed to evaluate the homogeneity of variances in C:M, AM, and CCD datasets, and Spearman’s rank correlation tests were conducted to assess associations between adrenal parameters and total body length (cm). Moreover, independent *t*-tests were used to compare C:M, AM and CCD between ‘acutely’ and ‘chronically’ stressed animals. To examine variation in AM across biological and physiological factors, a multifactorial analysis of covariance (ANCOVA) was conducted with sex (female, male), sexual maturity (immature, mature), and nutritional status (poor, moderate, good) as fixed factors. Since adrenal gland mass may be influenced by an individual’s size and age, total body length was used as a covariate to control for age-related effects. Differences in C:M ratios and CCD across the same categorical variables were assessed separately using multifactorial analyses of variance (ANOVAs).

The mass of each area (cortex, medulla, and “non-cortical/medullary tissue”) was estimated, and a multifactorial multivariate analysis of covariance (MANCOVA) was performed, incorporating sexual maturity as a fixed factor and body length as a covariate.

All statistical analyses were conducted using R Studio (Version 2023.03.1, RStudio, PBC, Boston, MA, USA) and Excel (Version 2022, Microsoft Corporation, Redmond, WA, USA). Data are presented as mean ± standard error (SE), and the level of statistical significance was set at *p* < 0.05.

### 2.4. Protein Analysis

#### 2.4.1. Tissue Preparation

A minimum sample size of 2 was used to test the protein extraction method of García-Vence et al. [43] as applied to FFPE tissues of the adrenal glands. These 2 samples were selected based on the integrity of the wax block and the sample, with priority given to those visually displaying a higher tissue mass. Adrenal gland sample 1 (named Adrenal 1) was obtained from a 194 cm mature female in poor nutritional condition, whose cause of death was bycatch (classified as acutely stressed). Adrenal gland sample 2 (named Adrenal 2) was retrieved from a 230 cm mature male, also in poor nutritional condition, that died from infectious disease (classified as chronically stressed). These contrasting cases were chosen to provide a preliminary comparison of protein profiles associated with different stress types.

Each adrenal slice was divided into approximately equal parts (estimated visually) and placed into separate microcentrifuge tubes (Fisher Scientific Ireland Ltd., Dublin, Ireland). While tissue sections were visually standardized in size, exact weights differed and were recorded prior to extraction to aid interpretation of protein yield.

Deparaffinization and protein extraction were carried out for both samples according to previous documented methods [43] with some modifications.

#### 2.4.2. Deparaffinization

Tissues were incubated with sufficient xylene to cover the sample in the microcentrifuge tubes (~1 mL) and heated for 5 min at 60 °C, followed by centrifugation at 10,000× *g* for 1 min at room temperature (RT). The supernatant was discarded, and this step was repeated with the pelleted adrenal tissue. 500 µL of 100% ethanol (EtOH) was added to resuspend the pelleted sample, followed by incubation for 1 min at RT. The sample was centrifuged at 10,000× *g* for 1 min at RT, following which the supernatant was discarded. The resuspension, incubation and centrifugation steps were repeated with decreasing concentrations of EtOH (90, 80 and 70%). Finally, the samples were incubated in 500 µL of de-ionized water (18 MΩ) for 30 min at RT. The resulting tissues were chopped into smaller pieces before the extraction.

#### 2.4.3. Protein Extraction

The buffer Tris-SDS-DTT-Glycine (62.5 mM Tris-Hydrochloride pH 6.8, 4% *w*/*v* Sodium Dodecyl Sulfate (SDS), 10% *v*/*v* glycerol, 100 mM Dithiothreitol (DTT)) was selected for the trial of this methodology [43]: 500 μL of buffer was added to each tissue sample, which was then boiled for 20 min at 100 °C, and incubated for 2 h at 60 °C. Samples were then sonicated for 30 min using a bath sonicator (ULTR, LBX Instruments, Barcelona, Spain) set at 4 °C, followed by centrifugation for 20 min at 15,000× *g* to remove tissue remnants. The supernatants were stored at −80 °C and quantified prior to digestion with a NanoDrop spectrophotometer (Thermo Fisher Scientific Ireland Ch Ltd., Dublin, Ireland) at a wavelength of 280 nm.

#### 2.4.4. One Dimensional SDS-PAGE

One-dimensional Sodium Dodecyl Sulfate-Polyacrylamide Gel Electrophoresis (SDS-PAGE) was performed as per Laemmli [50] to assess the quality, molecular mass and relative quantity of proteins present within the samples prior to digestion and mass spectrometry analysis. 20 μL of each extraction together with 20 μL of 2X SDS-PAGE loading buffer were mixed and boiled for 5 min at 100 °C. For each extraction, 20–30 μL of the boiled sample was then loaded onto a 10% acrylamide/bis-acrylamide gel and run in an electrophoresis cell (Bio-Rad, Hercules, CA, USA) at a constant amperage of 150 mA per gel until the samples visually reached the lower edge. The gels were stained with 0.05% Coomassie Brilliant Blue R-250 in a methanol/acetic acid solution (40%/10%) for 1.5 h at RT, then destained overnight in 40% methanol and 7.5% acetic acid with gentle agitation [39]. Gels were visualized using the Gel Doc EZ Gel Documentation System (Bio-Rad, Hercules, CA, USA).

#### 2.4.5. Preparation of Protein Samples for Liquid Chromatography-Mass Spectrometry (LC-MS/MS)

##### Filter Aided Sample Preparation (FASP) Method

Protein extracted from Adrenal 1 was subject to the FASP procedure as described by Wiśniewski [51]. Prior to FASP, the extracted protein concentration of Adrenal 1 was diluted to 4 μg/μL using extraction buffer. The sample was then mixed with 200 μL of Buffer UA (2.4 g Urea, 500 μL 1 M Tris pH 8.9). Contaminants and salts were removed by repeated ultrafiltration through 10 kDa Vivacon centrifugal concentrators (Sartorius, Göttingen, Germany) at 12,000× *g* for 20 min at RT (×2). 100 μL 0.05 M of iodoacetamide (IAA) was then added and vortexed for 1 min followed by incubation for 20 min in the dark. The concentrators were centrifuged at 12,000× *g* for 20 min and the flow-through was discarded. Subsequently, 100 μL of UA was added (×2) to the filter unit and centrifuged again under the same conditions. 100 μL of 50 mM ammonium bicarbonate (ABC) was added and samples were centrifuged at 14,000× *g* for 10 min (×2). The filter units were then transferred to new collection tubes followed by the addition of 50 μL of 50 mM ABC containing trypsin (1 μg per 100 µg of protein sample). The sample was incubated at 37 °C for 18 h, after which it was centrifuged at 14,000× *g* for 10 min. The peptides were eluted in 40 μL of 50 mM ABC. A final addition of 5 μL 50% acetic acid was added to the eluted peptides to stop the reaction. Samples were kept at −20 °C until the Zip Tip^®^ protocol (Merck Millipore, Darmstadt, Germany) was performed as per the manufacturer’s instructions.

##### In-Gel Protein Digestion Method

Protein extracted from Adrenal 2 was subject to in-gel protein digestion. The sample was prepared for SDS-PAGE as detailed in Section 2.4.4. The electrophoresis was carried out only until the sample entered the gel-resolving phase. In-gel digestion was performed according to previous protocols [45]. Briefly, the selected band was excised and centrifuged to collect and remove excess water. The gel piece was destained by the addition of 100 μL of 100 mM ammonium bicarbonate/acetonitrile (1:1, *v*/*v*) and 500 μL of neat acetonitrile until fully destained and decreased in size, and then dried in a vacuum centrifuge (SpeedVac, Thermo Fisher Scientific, USA). The gel piece was rehydrated with 100 μL of trypsin buffer (13 ng/µL trypsin in 10 mM ammonium bicarbonate in 10% (*v*/*v*) acetonitrile) for 3 h. A further 50–100 μL of trypsin buffer was then added to cover the piece, and the sample was incubated at 37 °C overnight. Finally, 100 μL of gel extraction buffer (1:2 (*v*/*v*) 5% formic acid/acetonitrile) was added to the tube and incubated for 15 min at 37 °C. The gel sample was centrifuged at 10,000× *g* for 5 min at RT and the supernatant was removed to a new tube. The Zip Tip^®^ protocol (Merck Millipore, Darmstadt, Germany) was then performed per the manufacturer’s instructions, to concentrate and purify the sample for mass spectrometry.

#### 2.4.6. Protein Identification by LC-MS/MS and Data Analysis

Both digested protein samples (Adrenal 1: FASP method; Adrenal 2: in-gel digestion method) were sent for LC-MS/MS analysis to the Conway Proteomics Core-Mass Spectrometry Resource at University College Dublin. Samples were de-salted prior to analysis using EvoTips (EvoSep, Odense, Denmark) as per the manufacturer’s instructions. LC-MS/MS was performed on a Bruker TimsTOF Pro mass spectrometer (Bruker Daltonics, Bremen, Germany) connected to an Evosep One chromatography system (Evosep, Odense, Denmark). Peptides were separated on an 8 cm analytical C18 column (Evosep, 3 µm beads, 100 µm ID) using the pre-set 33-samples-per-day gradient on the Evosep One.

The Bruker TimsTOF Pro mass spectrometer (Bruker Daltonics, Bremen, Germany) was operated in positive ion polarity with TIMS (Trapped Ion Mobility Spectrometry) and PASEF (Parallel Accumulation Serial Fragmentation) modes enabled. The accumulation and ramp times for the TIMS were both set to 100 ms, with an ion mobility range (1/k_0_) from 0.62 to 1.46 Vs/cm^2^. Spectra were recorded over a mass range of 100–1700 *m*/*z*. The precursor (MS) intensity threshold was set at 2500, and the precursor target intensity at 20,000. Each PASEF cycle consisted of one MS ramp for precursor detection followed by 10 PASEF MS/MS ramps, resulting in a total cycle time of 1.16 s.

Raw data from the LC-MS/MS was processed using MaxQuant (version 2.4.1.0) [52]. MS/MS spectra were matched to the UniProt reference proteome database of the common bottlenose dolphin (*Tursiops truncatus*) (45,131 proteins; UniProt proteome ID: UP000245320; accessed on 25 May 2023) as well as the contaminants file from MaxQuant. All searches were performed with tryptic specificity allowing two missed cleavages. Mass spectra were searched using the default setting of MaxQuant, namely a false discovery rate (FDR) of 1% on the peptide and protein levels. For bioinformatics analysis, data obtained from MaxQuant were imported into Perseus software (v2.0.10.0) [53]. Reverse hits, contaminants, and peptides identified by site were filtered out. The processed data were exported to Microsoft Excel where the protein identifications were sorted according to their corresponding samples. Protein ontology classification was analyzed using the FunRich v3.1.4 software [54] with annotations retrieved from the UniProt database and grouped according to Gene Ontology (GO) biological processes.

## 3. Results

### 3.1. Histological Examination

#### 3.1.1. Biological Related Effects on Adrenal Gland Mass

**Figure 2:**
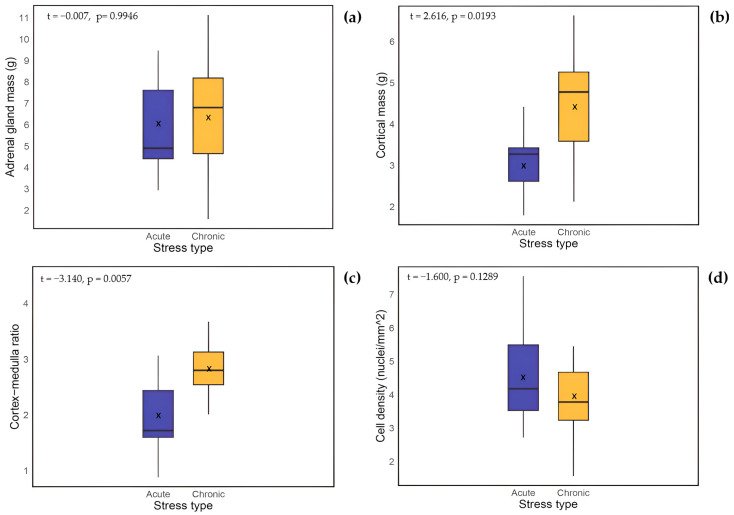
Boxplots displaying left adrenal parameters between acutely and chronically stressed common dolphins (*Delphinus delphis*). (**a**) Adrenal gland mass (*n* = 11, acute; *n* = 14, chronic); (**b**) Cortical mass (g) (*n* = 9, acute; *n* = 12, chronic; (**c**) Cortex-medulla ratio (*n* = 9, acute; *n* = 17,chronic); and (**d**) Cortical cell density (nuclei/mm^2^) (*n* = 11, acute; *n* = 13, chronic). Each boxplot displays the median (horizontal line), mean (× symbol), interquartile range and range of values. Group differences were assessed using Welch’s two-sample *t*-test (statistic and *p*-value are presented on each plot).

Of the 58 individuals, adrenal gland mass (AM) data were available for 51 dolphins and included within this analysis, excluding only those with missing measurements. No outliers were detected when analyzing boxplots. Paired *t*-tests compared left and right adrenal glands and showed no significant differences (*t* = 1.458, *p* > 0.1). Consequently, only left adrenals were used due to a larger sample size (*n* = 43). The mean left adrenal gland mass for acutely stressed animals was 5.925 ± 0.67 g (*n* = 11), while chronically stressed animals had a mean adrenal gland mass of 6.700 ± 0.76 g (*n* = 14) (see Table 2). No significant differences in mean values were observed between stress types (*t* = −0.007, *p* = 0.9946; Figure 2a). Additionally, the acute group (*n* = 11) showed a balanced distribution of nutritional status (25% poor, 50% moderate, 25% good), whereas the chronic group (*n* = 14) was predominately in poor condition (71% poor, 8% moderate, 8% good).

**Figure 3:**
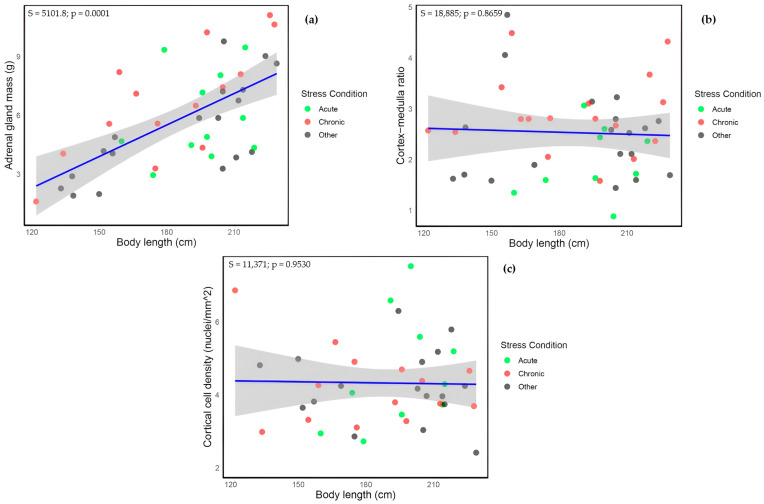
Relationship between left adrenal parameters and total body length. (**a**) Adrenal gland mass (g) plotted against total body length (cm); (**b**) Cortex-medulla ratio plotted against total body length (cm); and (**c**) Cortical cell density (nuclei/mm^2^) plotted against total body length (cm). Each panel shows the fitted trend line (in blue), with the shaded area representing the 95% confidence interval, the Spearman coefficient (S) and the *p*-value (p). Individual points are colored by stress condition: acute (green), chronic (red), and ‘other’ (gray).

A correlation analysis was carried out between AM and total body length (cm) (*n* = 43), revealing a highly significant relationship (S = 5101.8, *p* = 0.0001; Figure 3a). Thus, total body length (cm) was employed as a covariate in subsequent analyses. 

Because body length was a significant factor for AM and to account for biologically related effects, a factorial ANCOVA was performed including sex, sexual maturity, and nutritional status as categorical factors, and body length as covariate. No significant difference in AM between sexes (F = 1.050, *p* = 0.314) and nutritional status categories (F = 1.146, *p* = 0.332) was observed. In contrast, sexual maturity status showed a significant effect (F = 24.496, *p* < 0.001), with sexually mature individuals exhibiting, on average, a greater adrenal gland mass (7.26 ± 0.46 g; *n* = 24) compared to immature individuals (4.13 ± 0.39; *n* = 19). Since including the stress type category in the previous test substantially reduced sample size, a separate ANCOVA was conducted to compare adrenal mass across stress types, using body length as a covariate; again, no significant differences were found (F = 0.046, *p* = 0.832). Though for most chronically stressed individuals, AM was above the fitted trend line when compared against total body length, suggesting an enhanced physiological response (see Figure 3a). Body length distributions for individuals in each stress category are presented in Supplementary Figure S2. Notably, all animals classified as experiencing ‘acute’ stress measured over 160 cm in length.

#### 3.1.2. Biologically Related Effects on Cortex-Medulla Proportions

Histological examination of the adrenal glands revealed the characteristic cetacean structure previously described [18], featuring a zoned cortex surrounding a central medulla. A total of 56 samples were selected for C:M ratio analysis, excluding those with poor tissue quality that prevented accurate visualization of the full section. Following logarithmic transformation, all measurements and C:M ratios were normally distributed. No significant differences were found between the C:M ratios of the left and right glands (paired *t*-test: *t* = −0.168, *p* = 0.866); therefore, only left adrenals were included due to their larger sample size. Three outliers (C:M ratios of 5.500, 5.887 and 5.758), identified via boxplot analysis, were removed, resulting in a final sample of 45 glands for subsequent analyses. 

Spearman’s correlation analysis showed no significant association between the C:M ratio and total body length (S = 18,885, *p* = 0.866; Figure 3b). Further, no significant differences were detected when examining mean C:M ratios in relation to sex (F = 0.007, *p* = 0.932), sexual maturity (F = 1.701, *p* = 0.199), and nutritional status (F = 0.658, *p* = 0.523). In contrast, a comparison between stress types revealed that chronically stressed animals (2.892 ± 0.185) had significantly higher C:M ratios than acutely stressed animals (1.962 ± 0.231; *p* = 0.0057; Figure 2c).

**Figure 4:**
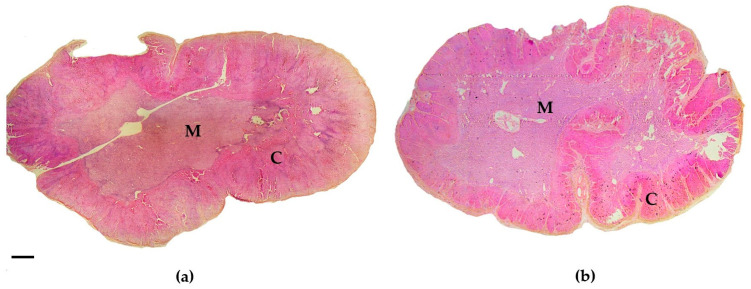
Cross-sections of the left adrenal glands from (**a**) a chronically stressed adult common dolphin (total body length: 228 cm) and (**b**) an acutely stressed adult common dolphin (total body length: 226 cm). Sections were overstained with hematoxylin and eosin. M, medulla; C, cortex. Scale bar: 1 mm.

In acutely stressed common dolphins, the left adrenal glands were composed of approximately 66% cortex and 31% medulla, with 3% categorized as “non-cortical/medullary tissue”—which included connective tissue, blood vessels, and the gland capsule. These proportions/percentages were determined by assessing the tissue type at each grid point. In contrast, chronically stressed animals exhibited a slightly higher percentage of cortex tissue (71%), compared to medulla (25%) and “non-cortical/medullary tissue” (4%) (Figure 4).

To distinguish between acutely and chronically stressed animals, the masses of the adrenal cortex, medulla, and “non-cortical/medullary tissue” were estimated by multiplying the total adrenal gland mass by the previously calculated percentage for each region. Chronically stressed animals (*n* = 17) had a mean cortical mass of 4.74 ± 0.50 g, mean medullary mass of 1.90 ± 0.22 g, and mean “non-cortical/medullary tissue” mass of 0.22 ± 0.07 g. In comparison, acutely stressed animals (*n* = 9) had a mean cortical mass of 3.14 ± 0.25 g, mean medullary mass of 1.85 ± 0.35 g, and mean “non-cortical/medullary tissue” mass of 0.16 ± 0.05 g. An independent *t*-test comparing adrenal cortical mass between acutely stressed (*n* = 9) and chronically stressed (*n* = 17) animals revealed a significant difference (*t* = 2.613, *p* = 0.019, Figure 2b). Finally, a multivariate analysis of covariance (MANCOVA) was conducted with the normally distributed masses of the adrenal cortex, medulla, and “non-cortical/medullary tissue” as dependent variables, stress group (acute vs. chronic) and sexual maturity as fixed factors, and total body length as a covariate. The analysis indicated no significant effect of stress group (F = 1.37, *p* > 0.1), sexual maturity (F = 2.37, *p* > 0.1), or total body length (F = 0.25, *p* > 0.8) on the combined dependent variables, suggesting that the significant difference in cortical mass between stress types identified by the *t*-test appears to be accounted for by other factors, such as total body length. However, the more limited statistical power of the MANCOVA, due to the small sample size, also needs to be considered.

#### 3.1.3. Biologically Related Effects on Cortical Cell Density

Secretory cell density in adrenal cortex cross-sections was assessed by counting the number of nuclei and dividing by the image area (in mm^2^) in the adrenal glands of 56 common dolphins. Samples that were too decomposed to allow accurate identification and counting of nuclei were excluded from the analysis.

The CCD data followed a normal distribution after logarithmic transformation, and a paired *t*-test revealed no significant differences between the left and right adrenal glands (*p* = 0.921). Consequently, only the left adrenal glands were used for further analysis. Three outliers identified via boxplot analysis were removed (CCD points: 9.31, 8.71, and 8.91 nuclei/mm^2^), and considered as exceptions, resulting in a final sample size of 41 glands.

Spearman’s correlation analysis showed no significant relationship between CCD and total body length (*n* = 41, S = 11,371, *p* = 0.953; Figure 3c). No significant differences were observed in mean CCD when considering sex (F = 0.349, *p* = 0.559), sexual maturity status (F = 1.162, *p* = 0.289) and nutritional status (F = 1.043, *p* = 0.364). Although the mean CCD was slightly higher in acutely stressed animals (5.01 ± 0.598 nuclei/mm^2^; *n* = 11) compared to chronically stressed animals (4.03 ± 0.210 nuclei/mm^2^; *n* = 13), this difference was also not statistically significant (Welch two sample *t*-test: *p* = 0.129; Figure 2d).

### 3.2. Protein Extraction

#### 3.2.1. Protein Yield and Quality of the Extraction Method

For the present study, two FFPE adrenal gland tissue sections, referred to as Adrenal 1 and Adrenal 2, from animals that died from incidental capture and infectious disease, respectively, were selected for proteomic analysis. The initial tissue weight, final protein concentrations (measured using a NanoDrop spectrophotometer (Thermo Fisher Scientific Ireland Ch Ltd., Dublin, Ireland)), and the documented cause of death for each sample are summarized in Table 3.

To assess the efficiency and consistency of protein recovery, SDS-PAGE was performed using a 10% acrylamide gel for both extracts. A fixed volume of each sample was loaded to enable a direct visual comparison of protein banding patterns. Gels were stained with Coomassie Brilliant Blue R-250 (Bio-Rad Laboratories, Hercules, CA, USA), and protein profiles were visualized using the Gel Doc EZ system (Bio-Rad Laboratories, Hercules, CA, USA). As shown in Supplementary Figure S1, a clear difference in band intensity was observed between the two samples. Adrenal 2 displayed a denser banding pattern than Adrenal 1, reflecting the higher protein yield obtained from this sample. These results visually confirm that both the deparaffinization and protein extraction protocols were successful and highlight variability in protein recovery that may be attributable to differences in extraction method, tissue preservation, or sample composition.

#### 3.2.2. Mass Spectrometry (LC-MS/MS) Analysis for Protein Identification

Following analysis of the MS/MS data, a clear difference in protein yield was observed between the two extraction methods. Adrenal 1, extracted using the FASP protocol, resulted in the identification of 22 proteins. In contrast, Adrenal 2, processed with the in-gel digestion method, yielded a total of 136 proteins, representing more than a six-fold increase. This difference likely reflects both methodological efficiency and potential variability in tissue quality or protein concentration. The full protein lists for each sample are provided in Supplementary Table S1 (Adrenal 1) and Supplementary Table S2 (Adrenal 2). Despite the disparity in total protein numbers, seven proteins were found in common between both adrenal samples, indicating possible core protein expression in dolphin adrenal tissue. The biological processes associated with the shared proteins identified in the samples are presented in Table 4. These include processes related to calcium-mediated signaling, iron metabolism, cytoskeletal regulation, and receptor signaling pathways.

**Figure 5:**
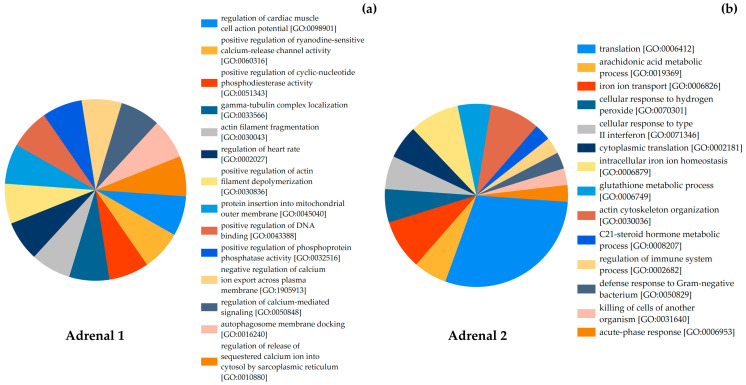
Functional classification of proteins identified from dolphin adrenal glands using FunRich software. (**a**) Biological processes associated with the 22 proteins identified in Adrenal 1, extracted using the Filter-Aided Sample Preparation (FASP) method. (**b**) Biological processes associated with the 136 proteins identified in Adrenal 2, extracted using the in-gel digestion method. Only the more abundant and stress-relevant biological processes are shown.

The functional classification of proteins performed with the FunRich v3.1.4 software [54] allowed the categorization of identified proteins according to their associated biological processes, and the most frequently enriched categories for each sample are presented in Figure 5.

In Adrenal 1, the proteins were associated with processes such as calcium release and transport, calcium-mediated signaling, and regulation of intracellular ion flow. Two biological process terms referred to cardiac muscle cell action potential and heart rate regulation, which were retained as part of the original GO annotations. Additional biological processes identified included actin filament fragmentation, tubulin localization, DNA binding, membrane docking, and protein insertion into membranes (Figure 5a).

For Adrenal 2, the functional classification revealed a broader distribution of biological processes. These included categories such as translation, iron ion transport, iron ion homeostasis, actin cytoskeleton organization, general metabolic processes, and immune-related responses (Figure 5b).

## 4. Discussion

This study provides new insights into the adrenal stress response in common dolphins through combined histological and proteomic approaches. By comparing individuals that died under conditions associated with either acute or chronic stress, we sought to characterize adrenal gland morphology and assess the feasibility of extracting and identifying proteins from archived formalin-fixed, paraffin-embedded tissues.

### 4.1. Histological Examinations

Gross and histological assessments of the available adrenal gland tissues showed no abnormal lesions such as tumors or cysts. In the analysis of adrenal gland mass, body length was included as a covariate to account for age-related variation, as cetacean organs and glands are known to grow throughout life, with growth rates gradually slowing after attainment of sexual maturity [55,56]. Our findings revealed no significant differences in average left adrenal gland mass between acute and chronic stress groups, diverging from earlier work reporting significant glandular enlargement in chronically stressed cetaceans [18]. Although adrenal mass was slightly higher in chronically stressed dolphins, on average, the difference from acutely stressed individuals was not substantial, and may be attributable to variation in age structure across groups, with body length serving as a proxy indicator of age (Supplementary Figure S2).

In cetaceans, Clark et al. [18] reported increased thickness of the adrenal cortex relative to the medulla in bottlenose dolphins with signs of chronic disease, while Kuiken et al. [16] similarly noted focal cortical enlargement in harbor porpoises (*Phocoena phocoena*) associated with chronic stress pathology. These changes were attributed to hypertrophy and/or hyperplasia of the zona fasciculata, which are processes that reflect prolonged ACTH exposure. Importantly, such remodeling may not affect medullary or capsular regions, potentially explaining the lack of significant difference in total adrenal mass despite significant cortical expansion.

It is also possible that chronic stress, when compounded by malnutrition, could lead to a net reduction in adrenal gland mass due to tissue depletion, despite localized hypertrophy in the adrenal cortex. In contrast, acutely stressed common dolphins, many of whom were in relatively good nutritional condition, may retain larger adrenal glands overall. As seen in our dataset, the chronic group was predominately in poor condition, while the acute group showed a more balanced distribution. This distinction is important, as body mass tends to decline with worsening nutritional condition. As noted earlier, a decline in the overall nutritional condition of common dolphins in the region has been observed, alongside an increase in strandings involving emaciated individuals or those that died from starvation, as well as infectious disease [12,41]. In a recent analysis of the same common dolphin sample employed in the current study, the scaled-mass index (SMI) values were highest in individuals in good condition and progressively lower in moderate and poor nutritional status categories, reflecting reduced body mass relative to length [41]. Severe emaciation and catabolism likely masked stress-related enlargement in chronically stressed animals, potentially explaining why total adrenal mass did not differ significantly between groups despite cortical hypertrophy. Despite this, adrenal enlargement was still evident in chronically stressed animals, suggesting that prolonged ACTH stimulation outweighed the catabolic effects of poor nutritional status. This interpretation is reinforced by our finding that chronically stressed individuals also had significantly higher cortical mass and cortex-to-medulla ratios, consistent with hypertrophy of the zona fasciculata described in previous odontocete studies [16,18]. Taken together, these results confirm that adrenal cortical measures provide a reliable post-mortem indicator of chronic stress in common dolphins. Additionally, body length and sexual maturity were significant predictors of adrenal mass, consistent with expected developmental growth patterns in cetaceans [55,56,57].

Microscopically, the significantly higher C:M ratio and cortical mass in chronically stressed individuals was reflected by a visibly thickened cortex relative to the medulla (Figure 4a). The hypertrophy interpretation is supported by our finding of lower cortical cell density in chronically stressed dolphins, despite their thicker cortices—consistent with enlargement of cells rather than increased cell numbers. Although the difference in cell density was not statistically significant, the pattern aligns with stress-related hypertrophy described in other cetaceans [58]. These results suggest that cortex-specific measures such as the C:M ratio and histological cellular analysis provide a more sensitive and reliable means of distinguishing chronic stress than adrenal gland mass.

Contrary to some earlier findings on harbor porpoises [16], acutely stressed common dolphins displayed slightly higher cortical cell densities than those experiencing chronic stress. Although not significant, this may reflect early adaptive responses to acute stress, or simply the absence of long-term remodeling in animals that died suddenly, thereby retaining a more typical cellular profile. Importantly, tissue decomposition and preservation quality may have also influenced nuclear counts, highlighting the limitations inherent in analysis of post-mortem material. Future studies incorporating cell-type-specific markers or immunohistochemical approaches could help distinguish whether changes in cell density reflect true hypertrophy, hyperplasia, or shifts in adrenal cell populations.

Other limitations must also be acknowledged. The comparative sample sizes for histological analysis between chronically (*n* = 14 to 17) and acutely (*n* = 9 to 11) stressed dolphins were relatively small, limiting statistical power and the ability to generalize findings across the broader population. This constraint is particularly relevant when interpreting subtle histological differences, such as cortical cell density, which may be influenced by individual variation or preservation quality. Furthermore, while our findings align with established patterns of adrenal response to chronic stress, larger datasets such as those used in previous studies [18] are needed to fully validate these trends. Nonetheless, this work provides a foundational framework for future investigations, particularly where access to larger, well-preserved post-mortem samples is available.

### 4.2. Protein Extraction and Functional Interpretation

This study demonstrates, for the first time, the feasibility of extracting and identifying proteins from FFPE adrenal gland tissues of common dolphins. Similar applications of FFPE material have shown success in both biomedical and wildlife research, including protein N-termini profiling in mice [59], proteomic workflow optimization [60], and protein stability in long-term clinical archives [61]. In conservation contexts, FFPE tissues have also enabled transcriptomic analysis in lampreys, demonstrating the method’s relevance for wildlife health monitoring [62]. 

Although FFPE preparation is known to induce protein crosslinking and reduce recovery efficiency [63], our protocol yielded sufficient material for both SDS-PAGE visualization and LC-MS/MS analysis. We acknowledge that formalin-induced crosslinking may also lead to peptide modification and reduced proteomic resolution, particularly for low-abundance or labile proteins [43,64]. However, the successful identification of proteins in our study demonstrates that, despite these limitations, FFPE-derived samples can still yield meaningful proteomic data, especially when optimized protocols and high-sensitivity detection platforms are used. This is a valuable step given that many marine mammal tissue repositories rely on paraffin embedding for long-term preservation, and fresh or frozen samples are often unavailable.

A clear difference emerged between the two extraction methods tested. The in-gel digestion approach outperformed FASP, producing over six times more identified proteins and yielding a more diverse protein profile. It is likely that this discrepancy may reflect differences in tissue quality between samples, and is no doubt affected by difference in protein concentration between the samples. However, the vast difference between the total number of proteins identified (22 versus 136) would tentatively suggest that in-gel digestion is a more effective strategy for proteomic work with cetacean FFPE adrenal glands. We acknowledge that differences in initial tissue mass, protein concentration, and preservation quality between the two adrenal samples may have contributed to this disparity. A systematic comparison of both methods is required before a definitive conclusion can be drawn.

While protein yields were modest compared to fresh tissue studies, the functional enrichment patterns observed in Adrenal 1 (acute stress, bycatch) and Adrenal 2 (chronic stress, infectious disease) were distinct and biologically meaningful. Establishing baseline protein concentrations in fresh adrenal tissue will be essential for validating FFPE-derived profiles and strengthening future interpretations. Among the proteins identified, only seven were common to both adrenal samples (Table 4), despite the large difference in total protein yield between extraction methods. Their associated biological processes—such as calcium-mediated signaling, intracellular iron ion homeostasis, cytoskeletal regulation, and receptor signaling—are tightly linked to adrenal gland function and stress responsiveness. Calcium signaling (Calmodulin-1) and cytoskeletal remodeling (Keratin and Actin) are critical for catecholamine release during acute stress [65], while iron metabolism (Hemoglobin subunit beta) and receptor pathways (Retinal dehydrogenase 1) may reflect more general cellular stress responses or immune activity [66]. The fact that these proteins were identified across both extraction methods suggests they may represent stable and constitutively expressed components of adrenal tissue, potentially serving as useful baseline references in future proteomic analyses of cetacean adrenal glands.

In Adrenal 1, representing an acutely stressed dolphin, only 22 proteins were identified, but these were disproportionately enriched in pathways related to calcium fluxes, cytoskeletal regulation, and cardiovascular modulation (Figure 5a). Notable processes included regulation of calcium-mediated signaling, release of sequestered calcium into the cytosol, and regulation of cardiac muscle cell action potential. These pathways are consistent with the acute activation of the sympatho-adrenal system, where rapid calcium-dependent catecholamine release from chromaffin cells is required to sustain “fight-or-flight” responses [65]. The enrichment of proteins linked to actin filament fragmentation and depolymerization further reflects cytoskeletal remodeling that supports vesicular trafficking and secretion [67]. Similar calcium-driven and cytoskeletal pathways have been described in rodent adrenal studies as early biomarkers of acute stress reactivity [68,69]. Interestingly, additional enrichment of mitochondrial insertion and autophagosome docking pathways may reflect cellular responses to hypoxia and ischemia, which are plausible in bycaught dolphins subjected to entanglement and asphyxia [70]. Together, these findings suggest that acute stress proteomes are dominated by fast-response, secretory, and metabolic processes, rather than structural remodeling.

By contrast, Adrenal 2, obtained from a chronically stressed dolphin with infectious disease, yielded a far richer proteome (136 proteins). Here, the dominant biological processes reflected oxidative stress regulation, immune activity, metabolic adaptation, and steroidogenesis (Figure 5b). Glutathione metabolism and cellular responses to hydrogen peroxide indicated oxidative stress, consistent with chronic disease states where reactive oxygen species accumulate [71]. Processes such as intracellular iron ion homeostasis and transport suggest systemic metabolic imbalance, while the acute-phase response and type II interferon response point to immune activation linked to prolonged infection [72]. Critically, enrichment of the C21-steroid hormone metabolic process highlights sustained adrenal cortical activity, with implications for glucocorticoid and mineralocorticoid production under persistent ACTH stimulation [73]. This finding supports our histological evidence of cortical hypertrophy and elevated C:M ratios in chronically stressed dolphins. Previous studies in cetaceans have reported altered steroidogenic profiles in stranded whales, including shifts in corticosterone, aldosterone, and catecholamine levels under extreme stress [74]. Similarly, molecular adaptations involving glucocorticoid receptor regulation and stress proteins such as HSP70 have been noted in cetacean adrenal tissues under chronic stress and contaminant exposure [6,75]. Our results provide proteomic-level evidence that such processes may be detectable in FFPE samples and could serve as biomarkers of chronic stress and immune activation in dolphins.

Nevertheless, several limitations must be acknowledged. The total number of proteins identified was modest compared to studies using fresh or frozen tissues [76], and only a small fraction of proteins overlapped between the two samples. This likely reflects both the constraints of FFPE preservation and the variability in tissue condition at necropsy. Additionally, the small sample size (*n* = 2) precludes generalization. Also, as with many studies of samples collected opportunistically from necropsied animals, controls from healthy animals are absent. Despite these constraints, the present work provides proof of concept that proteomic investigation of dolphin adrenal glands is achievable from archival material.

Future studies with larger sample sizes should aim to validate these candidate pathways, expand reference protein databases for cetaceans, and integrate proteomic data with endocrine markers such as blubber or skin cortisol [8,9].

### 4.3. Broader Implications and Future Perspectives

Our findings contribute to a growing body of evidence that chronic stress in dolphins manifests as subtle but detectable alterations in adrenal gland structure, particularly in cortex-to-medulla proportions. This supports the use of adrenal histology as a post-mortem tool for distinguishing between acute and chronic stress exposure in stranded cetaceans. Importantly, our work also establishes a methodological foundation for proteomic investigations in archival tissues, which could help identify reliable biomarkers of stress. Such biomarkers would complement existing tools, such as blubber cortisol [7,9], skin cortisol [8], or nutritional indices like ventral blubber thickness [7], offering a more integrated picture of cetacean health. 

Recent large-scale analyses further highlight how multiple stressors interact to compromise common dolphin health, showing that both chemical pollution (particularly PCBs) and the potential indirect effects of rising sea surface temperatures significantly increase the risk of infectious disease mortality in common dolphins [12]. Additionally, persistent organic pollutants (POPs) and heavy metals have been shown to disrupt HPA axis function, affecting glucocorticoid, aldosterone, and dehydroepiandrosterone (DHEA) production, with implications for long-term physiological regulation and health outcomes [77]. In parallel, structured necropsy-based datasets have proven useful for quantifying pathology and identifying system-level vulnerability to stressors, offering a scalable, objective tool for monitoring population health beyond simple body condition indices [78]. These findings reinforce that chronic stress signatures observed in stranded individuals cannot be interpreted in isolation, but rather as part of a wider context where anthropogenic and environmental pressures act synergistically to weaken immune responses and increase mortality risk.

Further work should refine extraction protocols to improve protein yield and consistency across samples, validate candidate protein biomarkers across larger datasets, and integrate histological, endocrine, and proteomic data. Such a multidisciplinary approach will improve our ability to assess both acute and chronic stress in wild dolphin populations, an urgent conservation need given ongoing threats from fishery bycatch, noise disturbance, prey depletion, and pollutant exposure [14,34].

## 5. Conclusions

This study shows that integrating histological and proteomic analyses of adrenal glands offers a valuable approach to assessing chronic stress responses in common dolphins. Chronically stressed individuals exhibited larger adrenal cortices and higher C:M ratios, consistent with cortical hypertrophy described in previous cetacean studies. Our findings suggest that cortex-specific metrics, particularly cortical mass and C:M ratios, provide a more reliable means of distinguishing chronic from acute stress than adrenal mass, which may be influenced by nutritional condition and other physiological variables. In addition, we conducted a pilot test of protein extraction from FFPE adrenal tissues. Although based on a very small sample size, the method successfully yielded biologically meaningful proteins, demonstrating the feasibility of applying proteomic analyses to archived material. Together, these results highlight the promise of combining tissue morphology and molecular data to develop robust biomarkers of stress in dolphins, supporting more accurate evaluations of health and resilience in wild populations facing increasing anthropogenic and environmental pressures.

## Figures and Tables

**Table 1:** Distribution of biological factors across stress categories in short-beaked common dolphins (*Delphinus delphis*) (*n* = 58). This table summarizes the number of individuals classified under each biological factor (sex, sexual maturity, nutritional status, and decomposition state) and their respective categories (e.g., male, female, mature, immature, etc.) within three defined stress groups: Chronically Stressed, Acutely Stressed, and “Other”. Totals for each biological level are provided in the rightmost column, while totals for each stress category appear in the bottom row.

Biological Factor	Biological Level	Chronically Stressed (*n*)	Acutely Stressed (*n*)	“Other” (*n*)	Total (per Level)
Sex	Male	8	10	13	31
	Female	11	4	10	25
	Unkown	0	0	2	2
Sexual maturity	Mature	11	6	16	33
	Immature	8	7	8	23
	Unknown	0	1	1	2
Nutritional status	Poor	12	3	10	25
	Moderate	5	7	13	25
	Good	2	4	2	8
Decomposition state	Fresh	7	8	8	23
	Slight	5	3	13	21
	Moderate	7	3	4	14
Total (per Stress type)		19	14	25	58

**Table 2:** Summary statistics for left adrenal gland mass, cortex-to-medulla ratio, and cortical cell density in acutely stressed dolphins, chronically stressed dolphins, stressed individuals (acute and chronic), and all individuals (acute, chronic, and other). For each group, the table reports the mean, median, standard error of the mean (SEM), interquartile range (IQR), and sample size (*n*) for each parameter.

Parameter	Statistics	Acutely Stressed	Chronically Stressed	All Individuals
Adrenal gland mass (g)	Mean	5.925	6.700	5.881
	Median	4.900	6.810	5.580
	SEM	0.677	0.760	0.391
	IQR	3.195	3.527	3.690
	n	11	14	43
Cortex-medulla ratio	Mean	1.962	2.892	2.529
	Median	1.720	2.801	2.573
	SEM	0.231	0.185	0.129
	IQR	0.840	0.586	1.092
	n	9	17	45
Cortical cell density (nuclei/mm^2^)	Mean	5.006	4.030	4.330
	Median	4.298	3.800	4.180
	SEM	0.598	0.210	0.180
	IQR	2.494	1.348	1.260
	n	11	13	41

**Table 3:** Weight (g), protein concentration (mg/mL), cause of death and stress type (acute, chronic) of adrenal gland sections (Adrenal 1 and Adrenal 2) used for protein extraction protocol.

Adrenal Gland	Weight (g)	Protein Concentration (mg/mL)	Cause of Death	Stress Type
Adrenal 1	0.176	5.878	Bycatch	Acute
Adrenal 2	0.265	16.218	Infectious disease	Chronic

**Table 4:** Proteins detected in common between FFPE-derived dolphin adrenal gland samples (Adrenal 1 and Adrenal 2) analyzed by LC-MS/MS and classified based on UniProtKB Gene Ontology (GO) annotations.

Uniprot ID	Protein Name	Biological Process
P18990	Hemoglobin subunit beta	Iron ion transport [GO:0006826]
A0A2U3V018	Calmodulin-1	Regulation of calcium-mediated signaling [GO:0050848] Positive regulation of ryanodine-sensitive calcium-release channel activity [GO:0060316] Regulation of release of sequestered calcium ion into cytosol by sarcoplasmic reticulum [GO:0010880] Regulation of cardiac muscle contraction [GO:0055117] Regulation of cardiac muscle cell action potential [GO:0098901]
A0A6J3S6J4	Keratin	Regulation of cytokinesis [GO:0032465]
A0A6J3PXY9	Actin	Regulation of cytokinesis [GO:0032465]
B6VQP8	Ferritin	Intracellular iron ion homeostasis [GO:0006879]
A0A2U3V291	Cytochrome b5 type B	Positive regulation of phosphoprotein phosphatase activity [GO:0032516]
A0A2U4BAL8	Retinal dehydrogenase 1	Positive regulation of receptor signaling pathway via JAK-STAT [GO:0046427]

## Data Availability

Data will be made accessible upon reasonable request to co-author sinead.murphy@atu.ie.

## Supplementary Materials

The following supporting information can be downloaded at: https://www.mdpi.com/article/10.3390/ani15192924/s1, Figure S1: SDS-PAGE visualization of protein integrity from Adrenal 1 and Adrenal 2; Figure S2: Length distribution of common dolphins included in each adrenal analysis; Table S1: List of proteins identified in Adrenal 1 using the Filter-Aided Sample Preparation (FASP) method; Table S2: List of proteins identified in Adrenal 2 using the in-gel digestion method.
