# Supplementary material for: Histological and Proteomic Approaches to Assessing the Adrenal Stress Response in Common Dolphins (Delphinus delphis)
Source: Animals (Basel). 2025 Oct 9;15(19):2924. doi: 10.3390/ani15192924 (PMC12523369; doi:10.3390/ani15192924)
Supplement: Supplementary file 1 [file animals-15-02924-s001.zip › animals-3875399-supplementary.pdf]

Histological and Proteomic Approaches to Assessing the Adrenal Stress Response in Common Dolphins (*Delphinus delphis*)

Claudia Medina Santana [1,*], Orla Slattery [1,2], Jim O'Donovan [3], Sinéad Murphy [1,*]

[1] Marine and Freshwater Research Centre, Department of Natural Resources and the Environment, School of Science and Computing, Atlantic Technological University, Dublin Road, H91 T8NW, Galway, Ireland

[2] Department of Analytical, Biopharmaceutical and Medical Science, School of Science and Computing, Atlantic Technological University, Dublin Road, H91 T8NW, Galway, Ireland

[3] Department of Agriculture, Food and the Marine, Regional Veterinary Laboratory, Model Farm Rd, Cork, T12 XD51, Ireland

* Correspondence: sinead.murphy@atu.ie, S.M; clamedinasantana@gmail.com, C.M.S.

Table of Contents

1. Figure S1: SDS-PAGE visualization of protein integrity from Adrenal 1 and Adrenal 2.

2. Figure S2: Length distribution of common dolphins included in each adrenal analysis.

3. Table S1. List of proteins identified in Adrenal 1 using the Filter-Aided Sample Preparation (FASP).

4. Table S2. List of proteins identified in Adrenal 2 using the in-gel digestion method.

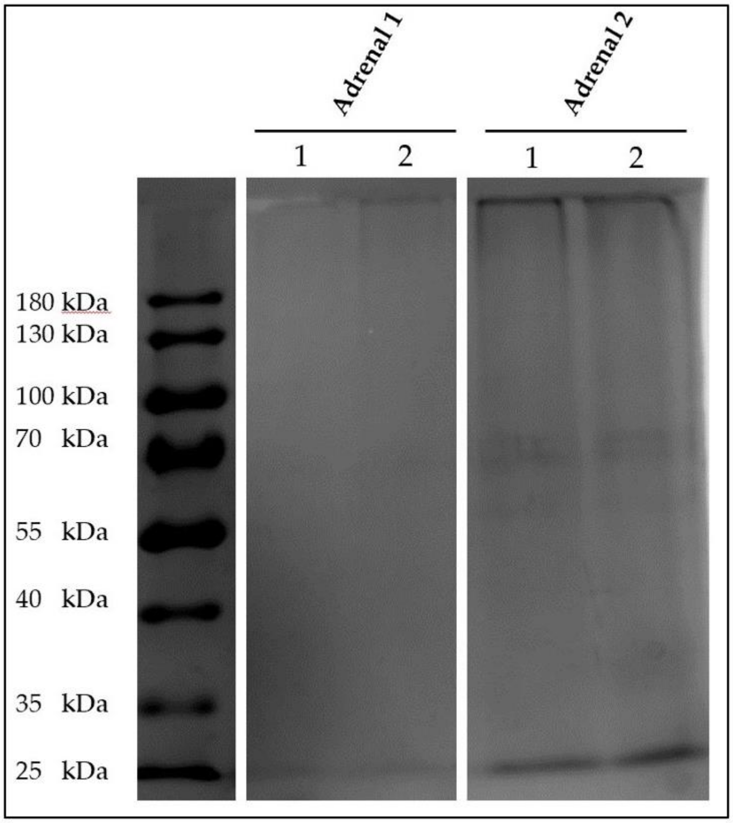

Figure S1. SDS-PAGE visualization of protein integrity from Adrenal 1 and Adrenal 2 using a one-dimensional 10% acrylamide/bis-acrylamide gel. Samples were run on the same gel under reducing conditions using a Tris-SDS-DTT-Glycine buffer system. The image shown here is a cropped composite derived from the original gel; non-relevant lanes have been removed for clarity, but all cropped sections originate from the same gel and run. A thin white dividing line indicates where the gel was cropped. Molecular weight markers are included. The original gel is provided as Supplementary File S1_Raw. All modifications comply with MDPI guidelines for gel image handling.

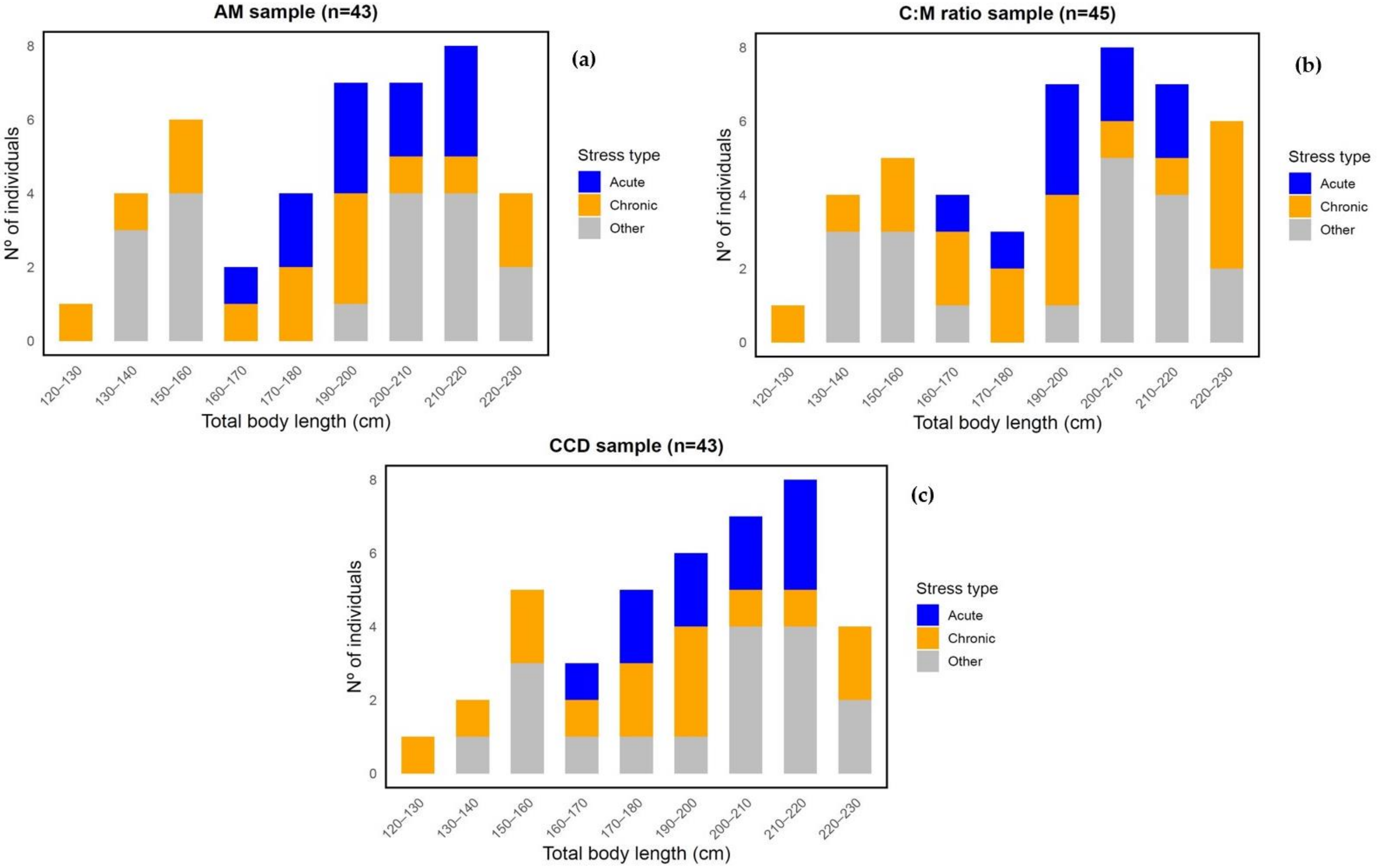

Figure S2. Length distribution (total body length in cm) of common dolphins included in each adrenal analysis.(a) Individuals included in the adrenal gland mass (AM) dataset (n = 43); (b) Individuals included in the cortex-to-medulla (C:M) ratio dataset (n = 45); (c) Individuals included in the cortical cell density (CCD) dataset (n = 43). In all panels, bar colors represent the stress classification assigned to each individual (acute, chronic, or 'other'), as indicated in the corresponding legends.

Table S1. List of proteins identified in Adrenal 1 using the Filter-Aided Sample Preparation (FASP) method followed by LC-MS/MS analysis. The table includes the UniProt ID and protein name for each of the 22 proteins identified. Protein identification was performed using MaxQuant software and matched against the *Tursiops truncatus* UniProt reference proteome.

Uniprot ID	Protein name
P18990	Hemoglobin subunit beta (Beta-globin) (Hemoglobin beta chain)
A0A2U3V018	Calmodulin-1 (Calmodulin-2) (Calmodulin-3)
A0A6J3S6J4	Keratin, type II cytoskeletal 5
A0A6J3PXY9	Actin, cytoplasmic 1
B6VQP8	Ferritin
A0A2U3V291	Cytochrome b5 type B
A0A2U4BAL8	Retinal dehydrogenase 1
A0A2U3V6U9	Mitotic-spindle organizing protein 1
A0A2U4ADF1	Proteasome subunit alpha type
A0A2U4AL99	Mitogen-activated protein kinase (EC 2.7.11.24)
A0A2U4AQ40	Destrin
A0A2U4AU67	Transcription intermediary factor 1-beta
A0A2U4BMR9	RNA helicase (EC 3.6.4.13)
A0A2U4C0L4	Mitochondrial import receptor subunit TOM22 homolog
A0A2U4C505	Histone H1.3
A0A2U4C845	Fetuin-B isoform X3
A0A6J3QW14	Leukocyte elastase inhibitor-like
A0A6J3S927	Heterogeneous nuclear ribonucleoprotein M isoform X11
A0A6J3RI42	Guanine nucleotide-binding protein subunit gamma
A0A6J3RMQ2	Prothymosin alpha isoform X2
A0A6J3RQQ7	Transmembrane protein 258 (Dolichyl-diphosphooligosaccharide-protein glycosyltransferase subunit TMEM258)
A0A6J3RR28	Cofilin-1

Table S2. List of proteins identified in Adrenal 2 using the in-gel digestion method followed by LC-MS/MS analysis. The table includes the UniProt ID and protein name for each of the 136 proteins identified. Protein identification was performed using MaxQuant software and matched against the *Tursiops truncatus* UniProt reference proteome.

Uniprot ID	Protein name
P18990	Hemoglobin subunit beta
A0A2U3V018	Calmodulin-1
A0A6J3S6J4	Keratin, type II cytoskeletal 5
A0A6J3PXY9	Actin, cytoplasmic 1
B6VQP8	Ferritin
A0A2U3V291	Cytochrome b5 type B
A0A2U4BAL8	Retinal dehydrogenase 1
A0A2U3V5B3	Annexin
A0A2U3V641	Superoxide dismutase [Cu-Zn] (EC 1.15.1.1)
A0A2U3V1W9	ATP synthase subunit beta (EC 7.1.2.2)
A0A2U3V2Z6	ATP synthase subunit alpha

A0A2U3V8D9	Peptidyl-prolyl cis-trans isomerase (PPIase) (EC 5.2.1.8)
A0A6J3RGW9	Lamin
P18978	Hemoglobin subunit alpha (Alpha-globin) (Hemoglobin alpha chain)
A0A2U3UYI4	Heat shock protein HSP 90-beta
A0A2U3V0C3	Vimentin
A0A2U3V5M2	Serum albumin isoform X1
A0A2U3V7G7	Proenkephalin-A
A0A2U4BNI3	10 kDa heat shock protein, mitochondrial
A0A6J3RYA6	LOW QUALITY PROTEIN: uncharacterized protein
A0A6J3QXM4	Chromogranin-A isoform X2
A0A6J3R7P0	ATP synthase F1 subunit delta
A0A2U3UZF5	Protein canopy homolog 2
A0A2U3UZZ2	Calnexin
A0A2U3V125	Ubiquitin-60S ribosomal protein L40
A0A2U4C5I8	Histone H2B
A0A2U3V6P6	Prohibitin
A0A2U4BJM6	Cytochrome b-c1 complex subunit 6, mitochondrial isoform X2
A0A2U3VA27	60S ribosomal protein L35a
A0A2U4A6P5	BTB/POZ domain-containing protein KCTD12
A0A2U4BA14	Tubulin alpha chain
A0A2U4BNH8	60 kDa heat shock protein, mitochondrial
A0A6J3Q4Z4	Retinol-binding protein
A0A2U4BUX4	Secretogranin-1
A0A2U4BWG2	Adrenodoxin, mitochondrial
A0A2U4BYC2	Malate dehydrogenase (EC 1.1.1.37)
A0A6J3Q630	LOW QUALITY PROTEIN: phosphatidylethanolamine-binding protein 1-like
A0A6J3QUH1	Thymosin beta
A0A6J3QLF7	NADPH:adrenodoxin oxidoreductase, mitochondrial (EC 1.18.1.6)
A0A6J3QR72	RNA-binding motif protein, X chromosome
A0A6J3QVI2	Pyruvate kinase (EC 2.7.1.40)
A0A6J3R7A7	40S ribosomal protein S28
A0A6J3RBF0	Collagen alpha-2(VI) chain isoform X1
A0A6J3RPP4	Neuroblast differentiation-associated protein AHNAK isoform X2
A0A6J3RTS7	Heat shock cognate 71 kDa protein
A0A6J3S7R6	phosphopyruvate hydratase (EC 4.2.1.11) (2-phospho-D-glycerate hydro-lyase)
A0A2U3UZY8	40S ribosomal protein S3 (EC 4.2.99.18)
A0A6J3S9W7	Mitochondrial import receptor subunit TOM6 homolog
A0A2U3V022	Enhancer of rudimentary homolog
A0A2U3V0A5	Proteasome subunit beta
A0A2U3V1P9	Aldehyde dehydrogenase, mitochondrial
A0A2U3V2D1	Tubulin beta chain
A0A6J3Q4P8	Dynein light chain roadblock
A0A2U4BL48	Nuclear migration protein nudC isoform X3
A0A2U3V3P5	Peroxiredoxin-2
A0A2U3V3V2	60S ribosomal protein L31
A0A2U4BID5	Heterogeneous nuclear ribonucleoprotein H isoform X15
A0A2U3V3Z7	40S ribosomal protein S18

A0A2U3V484	40S ribosomal protein S16 isoform X2
A0A2U3V496	Sodium/potassium-transporting ATPase subunit alpha
A0A2U3V4E5	Acylphosphatase (EC 3.6.1.7)
A0A2U3V4T2	Cytochrome P450 11B1, mitochondrial
A0A2U3V5V6	Crooked neck-like protein 1
A0A2U3V682	Epoxide hydrolase (EC 3.3.2.9)
A0A2U3V6D2	Protein disulfide-isomerase (EC 5.3.4.1)
A0A6J3S3U4	Thioredoxin-dependent peroxide reductase, mitochondrial-like isoform X2
A0A2U3V742	60S ribosomal protein L30
A0A2U3V7J0	Transgelin
A0A2U4CLU1	NSFL1 cofactor p47 (p97 cofactor p47)
A0A2U3V8R1	Dihydropteridine reductase
A0A2U3V921	Protein S100 (S100 calcium-binding protein)
A0A6J3RWS9	Heterogeneous nuclear ribonucleoproteins A2/B1
A0A6J3Q0B0	Elongin-B
A0A2U3V9J5	40S ribosomal protein SA (37 kDa laminin receptor precursor)
A0A2U3V9P7	Alpha-1-acid glycoprotein
A0A2U3VAH2	Glutathione peroxidase
A0A2U3ZYQ8	Citrate synthase
A0A2U4A0L5	Sm protein F
A0A2U4A670	Lysosome-associated membrane glycoprotein 1 isoform X2
A0A6J3QYH6	40S ribosomal protein S15a
A0A2U4A9H3	Cytochrome b-c1 complex subunit 2, mitochondrial
A0A2U4AA27	Eukaryotic translation initiation factor 2 subunit 1
A0A6J3RVK5	LOW QUALITY PROTEIN: voltage-dependent anion-selective channel protein 1-like
A0A6J3RVZ0	Histone H2A
A0A2U4AXQ1	Activated RNA polymerase II transcriptional coactivator p15 (SUB1 homolog)
A0A2U4AZU2	protein deglycase (EC 3.5.1.124) (Maillard deglycase) (Parkinsonism-associated deglycase)
A0A2U4B1B4	Stress-induced-phosphoprotein 1 isoform X2
A0A2U4B3A7	LOW QUALITY PROTEIN: heat shock 70 kDa protein 6
A0A2U4B3W1	SH3 domain-binding glutamic acid-rich-like protein
A0A6J3QSX6	Translationally-controlled tumor protein-like
A0A2U4BHA6	3-ketoacyl-CoA thiolase, mitochondrial isoform X3
A0A2U4BJK5	UMP-CMP kinase (EC 2.7.4.14) (Deoxycytidylate kinase) (CK) (dCMP kinase)
A0A2U4BMJ6	Carbamoyl-phosphate synthase [ammonia], mitochondrial
A0A2U4BMP4	Desmin
A0A2U4BN78	Long-chain specific acyl-CoA dehydrogenase, mitochondrial (EC 1.3.8.8)
A0A6J3RH28	Heterogeneous nuclear ribonucleoprotein K
A0A6J3S313	Dihydrolipoyl dehydrogenase (EC 1.8.1.4)
A0A2U4BYX2	Annexin
A0A2U4C084	Acylphosphatase (EC 3.6.1.7)
A0A2U4C2D3	40S ribosomal protein S21
A0A2U4CC96	Filamin-B isoform X8
A0A2U4CCR8	Bis(5'-adenosyl)-triphosphatase isoform X4
A0A2U4CE98	Dickkopf-related protein 3
A0A2U4CGD5	Transitional endoplasmic reticulum ATPase (EC 3.6.4.6)

A0A2U4CGI1	Moesin
A0A2U4CMG0	Transaldolase (EC 2.2.1.2)
A0A6J3PYN7	Vacuolar protein sorting-associated protein 16 homolog
A0A6J3Q6J3	Steroid 17-alpha-hydroxylase/17,20 lyase
A0A6J3Q7J5	Multifunctional fusion protein [Includes: Delta-1-pyrroline-5-carboxylate dehydrogenase (P5C dehydrogenase)
A0A6J3QF99	FXYD domain-containing ion transport regulator
A0A6J3QL47	Profilin
A0A6J3QPM4	Vesicle-associated membrane protein 2
A0A6J3QPT8	Migration and invasion enhancer 1
A0A6J3QQS2	LOW QUALITY PROTEIN: phenylethanolamine N-methyltransferase
A0A6J3QTV9	Filamin-A isoform X2
A0A6J3QTU6	B-cell receptor-associated protein (BCR-associated protein)
A0A6J3QTV5	Ferritin
A0A6J3QXD4	Heat shock protein HSP 90-alpha
A0A6J3QYE5	Sulfide:quinone oxidoreductase, mitochondrial isoform X3
A0A6J3R1R0	Cholesterol side-chain cleavage enzyme, mitochondrial (EC 1.14.15.6) (Cholesterol desmolase)
A0A6J3S9Q9	Stress-70 protein, mitochondrial (75 kDa glucose-regulated protein) (Heat shock 70 kDa protein 9)
A0A6J3R4R9	60S ribosomal protein L3-like
A0A6J3R5T9	Glucosidase 2 subunit beta
A0A6J3RAK3	Myosin light chain kinase, smooth muscle isoform X8
A0A6J3RC46	ATP synthase subunit e, mitochondrial
A0A6J3RPS2	Neutral alpha-glucosidase AB isoform X2
A0A6J3RS15	Glutathione S-transferase (EC 2.5.1.18) (GST class-pi)
A0A6J3RVV7	Aromatic-L-amino-acid decarboxylase
A0A6J3RXG2	Aldo-keto reductase family 1 member B1
A0A6J3S7M7	Keratin, type II cytoskeletal 8
A0A6J3S100	Estradiol 17-beta-dehydrogenase 8 isoform X4
A0A6J3S5A7	Myosin-9 isoform X2
A0A6J3S761	Myosin light polypeptide 6 isoform X3
A0A6J3S833	Parathymosin
B6VQQ0	Ferritin
B9UD79	Cytochrome c oxidase subunit 2
